# Does Patient Activation Reduce Mortality Risk in Patients Receiving Chronic Hemodialysis?

**DOI:** 10.34067/KID.0000000604

**Published:** 2024-11-21

**Authors:** Edward Zimbudzi

**Affiliations:** Monash Nursing and Midwifery, Monash University, Melbourne, Victoria, Australia; Department of Nephrology, Monash Health, Melbourne, Victoria, Australia; and School of Public Health and Preventive Medicine, Monash University, Melbourne, Victoria, Australia

**Keywords:** chronic dialysis, chronic hemodialysis, CKD, chronic kidney failure, chronic renal disease, hemodialysis adequacy, patient satisfaction, patient self-assessment, patient-centered care, lifestyle medicine

Optimal care occurs when patients possess the skills, knowledge, and confidence needed to effectively manage their own health, a concept known as patient activation.^1^ The interest in patient activation and its potential applications in nephrology care continues to grow, partly because of its incorporation into kidney disease legislative policy, especially in the United States.^2^ Although there is a robust evidence base of the benefits of patient activation, outcomes in the context of patients receiving chronic hemodialysis have not been extensively studied.

In the current issue of *Kidney360*, Gopal *et al.*^3^ evaluated a cohort of 925 prevalent patients receiving in-center hemodialysis among ten facilities from a single midsized provider, offering very important data regarding the association between patient activation and outcomes in patients receiving hemodialysis. This study investigated whether patient activation status can act as a marker for patient risk among those receiving chronic hemodialysis. This important step helps clinicians to proactively identify patients at highest risk of future health issues, allowing for personalized care and targeted interventions to improve activation. Patient activation was assessed with the Patient Activation Measure-13, a survey that is scored from 0 to 100, with higher scores indicating greater activation.^1^ Using this instrument, patients rate the degree to which they agree or disagree with each of the 13 statements that are about beliefs, confidence in the management of health-related tasks, and self-assessed knowledge. Findings revealed a lower mortality risk among patients with higher activation scores, which persisted even after adjusting for various factors traditionally associated with increased mortality. Notably, higher activation scores did not correlate with hospitalization or nonadherence markers.

The effect of patient activation on mortality has not been widely studied, especially in patients receiving chronic hemodialysis. However, other measures of self-care, such as medication nonadherence, are known to increase the risk of death in patients receiving chronic hemodialysis.^4^ The results of the present study point to a possible protective role of high patient activation against mortality. This is corroborated by a prospective study of older patients with heart failure, which showed that lower activation levels were associated with increased 30-day mortality after adjusting for key confounding variables, such as comorbidities and health literacy.^5^ This highlights the importance of patient activation not only in preventive health agenda, but in reducing the risk of mortality in patients with chronic diseases.

The study reports no association between patient activation and hospitalization. This is in contrast to prior studies that have demonstrated reduced risk of hospitalization among patients with high activation.^5,6^ A potential reason for this is that the health status of patients receiving chronic hemodialysis is often worse than in prior studies of people with chronic diseases, leading to unavoidable hospitalization, regardless of activation level. Interestingly, some studies have reported significant increases in hospitalizations among patients who received activation interventions.^7,8^ A possible explanation for this is that patients with high activation can confidently engage in their care and navigate the health care system, leading them to seek admission promptly if they feel unwell. While high activation can lead to increased hospitalization, there is a potential benefit in reduced length of hospital stay among patients who receive activation interventions.^9^ The possibility of reverse causality needs to be acknowledged when exploring the relationship between patient activation on hospitalization.

While this study provides valuable insights into the potential role of patient activation in reducing mortality among patients receiving chronic hemodialysis, it is important to highlight its potential limitations. The study examined interindividual differences in activation scores rather than intraindividual changes over time. In many instances, aggregated group data do not adequately reflect individual variability in behavior, potentially leading to conclusions that do not fully account for potential changes in patient activation during the study period. In addition, given the retrospective nature of the study that used electronic medical record data, potential variables that may attenuate the independent effects of patient activation on mortality were not adjusted for. These variables include comorbidities other than diabetes, health literacy, and frequency of depressive symptoms, which commonly affect patients receiving chronic hemodialysis. Finally, the Patient Activation Measure-13 survey has been validated extensively in various clinical settings and patient populations, including those with CKD,^10^ but not on dialysis. In the absence of reliable and validated kidney-specific instruments to assess activation in patients receiving chronic hemodialysis, the Patient Activation Measure survey is a justified option.

In conclusion, despite the study's strengths, such as its large size, data completeness, robust analysis, and strong clinical relevance, it still has critical limitations that need addressing. These limitations stem from the retrospective nature of the design and reliance on electronic medical records. This approach meant that critical data on important factors known to affect patients receiving chronic hemodialysis, such as commodities and health literacy, could not be collected. The results need to be interpreted in the context of these factors, which could have influenced the study's internal validity. Overall, the results are pivotal, marking a crucial step in understanding the role of patient activation in reducing mortality risk among individuals with chronic diseases.

This study opens up opportunities for future research to prospectively examine the relationship between patient activation and mortality in patients receiving chronic hemodialysis. Analysis across the four activation levels may also help to understand whether there is evidence of an inverse linear dose-response relation between patient activation and mortality in patients receiving chronic hemodialysis.
